# Alamandine: Potential Protective Effects in SARS-CoV-2 Patients

**DOI:** 10.1155/2021/6824259

**Published:** 2021-11-08

**Authors:** Ava Soltani Hekmat, Kazem Javanmardi

**Affiliations:** Department of Physiology, Fasa University of Medical Sciences, Fasa, Iran

## Abstract

Coronavirus disease 2019 (COVID-19) can occur due to contracting severe acute respiratory syndrome coronavirus 2 (SARS-CoV-2). COVID-19 has no confined treatment and, consequently, has high hospitalization and mortality rates. Moreover, people who contract COVID-19 present systemic inflammatory spillover. It is now known that COVID-19 pathogenesis is linked to the renin-angiotensin system (RAS). COVID-19 invades host cells via the angiotensin-converting enzyme 2 (ACE2) receptor—as such, an individual's susceptibility to COVID-19 increases alongside the upregulation of this receptor. COVID-19 has also been associated with interstitial pulmonary fibrosis, which leads to acute respiratory distress, cardiomyopathy, and shock. These outcomes are thought to result from imbalances in angiotensin (Ang) II and Ang-(1-7)/alamandine activity. ACE2, Ang-(1-7), and alamandine have potent anti-inflammatory properties, and some SARS-CoV-2 patients exhibit high levels of ACE2 and Ang-(1-7). This phenomenon could indicate a failing physiological response to prevent or reduce the severity of inflammation-mediated pulmonary injuries. Alamandine, which is another protective component of the RAS, has several health benefits owing to its antithrombogenic, anti-inflammatory, and antifibrotic characteristics. Alamandine alleviates pulmonary fibrosis via the Mas-related G protein-coupled receptor D (MrgD). Thus, a better understanding of this pathway could uncover novel pharmacological strategies for altering proinflammatory environments within the body. Following such strategies could inhibit fibrosis after SARS-CoV-2 infection and, consequently, prevent COVID-19.

## 1. Introduction

COVID-19 is an extremely contagious disease triggered by severe acute respiratory syndrome coronavirus 2 (SARS-CoV-2). The first case of COVID-19 occurred in December 2019 in Wuhan, China. The World Health Organization (WHO) declared it as a pandemic [1].

Coronaviruses comprise a single-stranded RNA enveloped in a capsid that is encapsulated in a spike (S) glycoprotein containing membrane. This S glycoprotein enables the virus to bind to host receptors, enter host cells, and initiate membrane fusion [2]. According to the literatures, angiotensin-converting enzyme (ACE) 2 is a specific functional receptor for SARS-CoV2 [3]. A type II transmembrane serine protease is the main host protease by which S glycoproteins are activated [4]. The viral entry of this protease can be inhibited by camostat mesylate [5].

According to Zhou et al., SARS-CoV-2 can infiltrate ACE2-expressing cells, whereas it cannot enter cells that do not have ACE2. Moreover, it cannot enter cells with conventional COVID-19 entry receptors such as aminopeptidase N and dipeptidyl peptidase 4 (DPP4) [6]. Such findings signify ACE2's status as the primary cell receptor for SARS-CoV-2 [7].

Regarding SARS-CoV-2 infections, in particular, ACE2 receptors (or their transmembrane domain) are internalized with the virus. S glycoproteins do not occlude the ACE2 receptor's catalytically active site [8]. Moreover, binding takes place regardless of ACE2's peptidase activity. Also, binding and membrane fusion appear to be promoted by a disintegrin and transmembrane protease serine 2 (TMPRSS2), metallopeptidase domain 17 (ADAM17), and TNF-converting enzyme, among other transmembrane proteinases and some proteins, including vimentin and clathrin [8].

Given the current absence of a specific treatment for COVID-19 which has high morbidity and mortality rates, it is vital to discover a novel drug for COVID-19 treatment.

The clinical characteristics of COVID-19 patients and the fact that ACE2 is a SARS-CoV-2 receptor suggest that SARS-CoV-2 infection causes an imbalance in the RAS [7]. In this review, we discuss the following: (i) different treatment options for COVID-19 patients and some clinical strategies for rebalancing the RAS. These treatments utilize ACE inhibitors and angiotensin receptor blockers, and they involve the activation of the RAS's protective arm with MasR agonist (Ang-(1-7)), (ii) ACE2-based treatments, and (iii) the protective effect of alamandine on COVID-19 patients, because of its anti-inflammatory, antifibrotic, and antioxidative properties.

The information provided in this study was derived from English language publications found in the PubMed and Scopus databases, as well as the Google Scholar search engine. The search terms were alamandine, RAS, ACE2, Ang-(1-7), COVID-19, SARS-CoV-2, and cytokine storm; we also searched for combinations of these terms.

## 2. Treatments for COVID-19

Although only one medication to treat COVID-19 has been approved by the U.S. Food and Drug Administration (FDA), many medications are being tested [9]. Below, we describe various available treatments for COVID-19 [9].

### 2.1. Antiviral and Antimalarial Drugs

Remdesivir is the only drug approved by the FDA to treat COVID-19 [9]. Remdesivir binds to the viral RNA-dependent RNA polymerase and inhibits viral replication by preventing RNA transcription from being completed. In *in vitro*, it exhibits anti-SARS-CoV-2 activity [9]. However, the results of remdesivir randomized controlled trials (RCTs) have been inconsistent. Current RCTs of remdesivir focused on hospitalized patients have revealed that remdesivir had no significant therapeutic effects for COVID-19 patients. However, several individual studies have shown promising outcomes among individuals at the early stages of the disease [9, 10].

Chloroquine and hydroxychloroquine are used to treat malaria, as well as several autoimmune disorders such as systemic lupus erythematosus and rheumatoid arthritis. Chloroquine and hydroxychloroquine prevent viral infection by increasing the pH (from approximately 4.0 to 6.0) of cellular endosomes (which is required for virus-cell fusion) and interfering with SARS-CoV cellular receptor glycosylation [11]. RCTs and meta-analyses indicate that chloroquine and hydroxychloroquine do not protect against SARS-CoV-2 infection, decrease mortality, reduce the duration of hospital stay, or improve clinical outcomes [9].

### 2.2. Corticosteroid

Patients with severe COVID-19 may have a systemic inflammatory response, leading to lung injury and multisystem organ failure [9]. However, the potent anti-inflammatory properties of corticosteroids may prevent or mitigate these undesirable effects. Corticosteroids may decrease the excessive inflammation in patients with severe cases of COVID-19, but they may also induce immunosuppression and exacerbate the infection. Furthermore, long-term and high-dose corticosteroid use may cause side effects, including avascular necrosis and hyperglycemia. Corticosteroids are often prescribed for severe patients who frequently suffer from acute respiratory distress syndrome and/or sepsis [9, 12].

### 2.3. Interleukin-6 Inhibitors

IL-6 is a proinflammatory cytokine produced by monocytes, fibroblasts, and lymphocytes, and SARS-CoV infection induces bronchial epithelial cells to generate IL-6 in a dose-dependent manner. Tocilizumab is an IL-6 receptor monoclonal antibody [9] that has been proposed to help protect against COVID-19. Initial studies on the use of tocilizumab as a COVID-19 treatment yielded conflicting results. The two largest randomized controlled tocilizumab trials (REMAP-CAP and RECOVERY) indicated that tocilizumab reduced the mortality rate among hospitalized patients, especially those needing oxygen due to an inflammatory response [9, 13]. Although IL-6 receptor antagonists appeared to help COVID-19 patients who need substantial amounts of oxygen, they are not yet suitable for general use in adults who have mild cases of the disease or require prolonged invasive mechanical ventilation [13].

### 2.4. Neutralizing Monoclonal Antibodies

Neutralizing monoclonal antibodies are a type of immunological molecule that can recognize and bind to specific antigens. Because large quantities of neutralizing monoclonal antibodies can be generated rapidly, they are considered a possible candidate for treating new infectious diseases. Specifically, they have been employed effectively to treat the Ebola virus disease [10]. Monoclonal antibodies against SARS-CoV-2 mainly target the S glycoprotein, thus inhibiting SARS-CoV-2 recognition and binding to the human ACE2 receptor. The result of this mechanism is viral cell entry [10].

The FDA has granted emergency use authorization (EUA) to many neutralizing monoclonal antibodies, including bamlanivimab, bamlanivimab with etesevimab, casirivimab with imdevimab, and sotrovimab [13]. Many neutralizing monoclonal antibody therapies seem to reduce viral load and prevent mortality in people at high risk of disease progression [7, 10, 13].

Considering rapid SARS-CoV-2 mutations, monotherapy with neutralizing antibodies may result in further mutational escape. Because SARS-CoV-2 variants are continuously developing, it is crucial to monitor the variant types and their susceptibilities to different antibody therapies [7].

## 3. The Renin-Angiotensin System

The RAS is an essential hormonal system for regulating blood pressure [14]. The RAS is a complex system of various peptides that carry out diverse biological actions mediated by specific receptor subtypes [15]. Angiotensin (Ang) II is a potent bioactive molecule generated from Ang I by an ACE. The classic RAS includes the ACE-Ang II-Ang II type 1 receptor (AT_1_R) axis. This axis ensures vasoconstriction and water intake, sodium retention, fibrosis, inflammation, and increased oxidative stress. The ACE2-Ang-(1-7)-alamandine pathway is the main component of the other (nonclassical) type of RAS. This RAS combats many of the actions performed by the Ang II-AT_1_R axis [15, 16].

Various cardiovascular diseases develop via the classic RAS [17]. In this system, collagen is synthesized, and fibroblast is proliferated in the heart via Ang II. Cardiac hypertrophy and fibrosis are common effects of this process, and they often lead to adverse ventricular remodeling [18]. Ang II is also commonly responsible for inflammatory processes and alterations in the electrophysiological properties of the heart [19].

AT1 receptor stimulation leads to vasoconstriction and, in turn, increased catecholamine release and aldosterone production [17]. These outcomes subsequently trigger fibrosis, inflammation, and decreases in collagenase activity and mitogen-activated protein kinase (MAPK) expression [20]. NADPH oxidase downregulation, decreased smooth muscle cell expression, and increased reactive oxygen species (ROS) production are vital components of AT1 receptors' proinflammatory processes. The activity of various proinflammatory transcription nuclear factors (e.g., nuclear factor-kappaB (NF-*κ*B)) is also increased [21]. Additionally, ATI receptors cause the release of proinflammatory cytokines, such as tumor necrosis factor-*α* (TNF-*α*) and interleukin-6 (IL-6) [19].

RAS blockers (ACE-inhibitors and II receptor blockers (ARBs)) are a potential component of treatment for COVID-19 patients. Research shows that these patients have high ACE2 levels, which correlate with high viral loads; therefore, ACE2 is likely linked to COVID-19 pathogenesis. Early evidence suggested that RAS blockers increase the severity of health outcomes due to increased ACE2 mRNA expression, which, in turn, promotes the virulence of SARS-CoV-2 [22]. However, no studies have confirmed this relationship. Moreover, no evidence supports the notion that ACE2 activity is linked to mortality due to SARS-CoV-2 [23].

Moreover, empirical studies have not confirmed the connections between ARB and ACE-inhibitor use and COVID-19 diagnosis, hospital admission due to COVID-19, and COVID-19 severity [19, 24].

Furthermore, research indicates that the use of RAS blockers and non-RAS blockers results in similar mortality rates among COVID-19 patients [25, 26]. Opinions and guidelines are inconsistent regarding whether medications that upregulate ACE2 levels should be continued, primarily because of the current lack of evidence on this matter [27].

## 4. The Protective Arm of RAS

**Figure 1:**
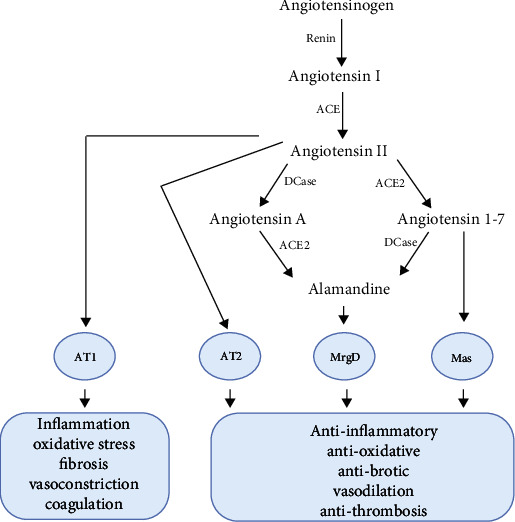
The renin-angiotensin system cascade. ACE: angiotensin-converting enzyme; ACE2: angiotensin-converting enzyme 2; ACE: angiotensin-converting enzyme; Dcase: decarboxylase.

Unlike the classic RAS, the nonclassic RAS protects the heart by reducing inflammation, fibrosis, and cardiac electrical remodeling. It also plays a role in vasodilation, hypertrophy, and thrombosis mitigation. Of note, alamandine and Ang-(1-7) belong to this protective arm of RAS [28]. This RAS axis has antioxidative, anti-inflammatory, and antifibrotic properties. Specifically, the ACE2/Ang-(1-7)/mitochondrial assembly receptor (MasR) and alamandine/MrgD axes might help protect the body against COVID-19 [29] (Figure 1).

## 5. Angiotensin-Converting Enzyme 2

ACE2 directly converts Ang II into Ang-(1-7). Among diabetes patients, serum and urinary ACE2 activity increases, as do hypertension and the risk of heart failure [15]. Whereas the circulating levels of ACE are generally high, ACE2 levels tend to be low. This pattern might indicate reduced shedding or perhaps a restricted vascular expression of peptidase [15]. Thus, ACE2 is a vital component of the RAS, owing to its capacity to metabolize Ang II. It also promotes the production of Ang-(1-7), thus activating the Ang-(1-7)-AT_2_/MasR axis.

Over time, the administration of soluble ACE2 can lower the risk of cardiac and renal injury, fibrosis, and inflammation in mice with type 1 and type 2 diabetes [15]. Furthermore, adipose tissue proinflammatory cytokines (IL-1*β*, TNF-*α*, IL-6) and iNOS were found to increase in ACE2 knockout mice [8].

When ACE2 activity is reduced or absent, Ang I tends to be converted into Ang II. At the same time, Ang II is not effectively converted into Ang-(1-7) or alamandine. Because of this, the levels of proinflammatory Ang II are increased, while the levels of anti-inflammatory Ang-(1-7) and alamandine are reduced [19].

Conceptually, using ACE2 to alter the balance of Ang II and Ang-(1-7) over time is a challenging process. Ang II levels can be lowered via exogenous ACE2, and this process is likely to generate Ang II due to the inactivation of renin's negative feedback mechanisms [15]. Therefore, it seems unlikely that high levels of ACE2 can reduce Ang II and increase Ang-(1-7) in the long term, except perhaps if an ACE inhibitor is also involved [15].

Almost every organ in the human body, including the lungs, heart, kidney, nasopharynx, smooth muscle cells, and testes, expresses different ACE2 receptor levels [30]. ACE2 is primarily expressed on type II alveolar epithelial cells within the respiratory system [8]. However, low levels of this receptor are also expressed in the oral and nasal mucosa and nasopharynx surfaces' epithelial cells. Such findings indicate that SARS-CoV-2 mainly targets the lungs [31].

Moreover, proximal tubule cells, bladder urothelial cells, and myocardial cells express high levels of ACE2. ACE2 receptors are also abundant in the enterocytes of the small intestine, especially in the ileum [32]. After infecting the lungs, SARS-CoV-2 can spread through the bloodstream to other organs with elevated ACE2 expression levels. For example, enteric symptoms were exhibited by as many as 67% of patients who developed diarrhea while infected with SARS, and a significant proportion of COVID-19 patients showed similar symptoms [31].

The soluble human recombinant ACE2 is useful for treating lung injuries caused by viral infections [32]. Thus, ACE2 could be used to treat COVID-19. This argument is based on the fact that the entry receptor of ACE2 mediates SARS-CoV-2 infections and that it impacts cardiac and lung damage [33] .ACE2 blockers can also prevent viruses from entering cells. Therefore, treatments involving ACE2 blockers could reduce ACE2's protective and anti-inflammatory activity, leading to a higher risk of lung damage.

As such, it could be desirable to block viral entry via protease inhibitors that target TMPRSS2 protease upon SARS-CoV-2 cell entry [31]. This alternative would eliminate the risk associated with endogenous ACE2 activity. Also, recombinant human ACE2 could improve the function of downregulated ACE2; subsequently, Ang-(1-7) and alamandine could return the functioning of the RAS to normal by diminishing the effects of high Ang II levels [19]. However, clinical trials using ACE2-based treatments are needed to ensure that such treatments present no adverse effects and that they increase the likelihood of positive outcomes in COVID-19 patients.

## 6. Angiotensin 1-7

Ang-(1-7) can bind to MasR and AT_2_R and subsequently counter the harmful effects imposed by the Ang II/AT_1_R axis. Ang-(1-7) has also been shown to have antiarrhythmic, antithrombotic, antihypertensive, and vasodilatory properties that protect the cardiopulmonary system. Furthermore, studies on animals indicate that low Ang-(1-7) levels are associated with acute respiratory distress syndrome (ARDS), while the upregulation of Ang-(1-7) appears to reduce the prevalence of reactive oxygen species and pulmonary fibrosis. By doing so, Ang-(1-7) prevents tissue damage. Beyond this, research has shown that the Ang-(1-7)/MasR axis exhibits anti-inflammatory effects by blocking the NF-*κ*B pathway and diminishing the prevalence of proinflammatory cytokines (e.g., TNF-*α* and IL-6) [33].

There are various Ang-(1-7) agonists, including cyclic Ang-(1-7), hydroxypropyl *β*-cyclodextrin (HP*β*CD)/Ang-(1-7), AVE-0991, CGEN-856, and CGEN-857 [33] that exhibit vasodilation and improved cardiac remodeling by binding to MasR. This binding activity allows these agonists to exert the same effects as Ang-(1-7) with high *in vivo* stability. However, there are still several Ang-(1-7) agonists that have been empirically investigated in humans—thus, there is not enough data regarding the safety of using some of these agonists for treating diseases.

The FDA has approved a pharmaceutical Ang-(1-7) formulation called TXA127 as an orphan drug used to treat various ailments, including pulmonary arterial hypertension and Duchenne muscular dystrophy [33]. Ang-(1-7) can increase the mRNA expression of extracellular signal-regulated kinase-1 (ERK)1/ERK2 [34]. Various MasR agonists have been studied to determine their capacity to treat cardiovascular diseases. One such agonist is the nonpeptide compound AVE 0991. This agonist, in combination with aliskiren (a renin inhibitor), was studied in rats with experimental hypertension; the results revealed decreased blood pressure [35]. Moreover, Ang-(1-7) has been reported to have a cardioprotective effect in murine models of heart failure, highlighted by reduced ejection fractions [15].

Preclinical evidence indicates the introduction of Ang-(1-7) improves oxygenation while reducing inflammation and fibrosis [15].

Ang-(1-7) might protect against COVID-19. Specifically, increases in the circulating levels of ACE2 [36] and Ang-(1-7) [37] among COVID-19 patients could be a protective mechanism. This notion is supported by Wang et al., who showed that a high Ang-(1-7)/Ang II ratio predicts favorable outcomes in patients with heart failure [38].

Several unpublished clinical trials are currently being conducted to determine the efficacy and safety of treating COVID-19 patients with Ang-(1-7) [19].  Table 1 shows the characteristics of these ongoing studies.

## 7. Alamandine

Alamandine is a recently discovered aspect of the RAS that protects the cardiovascular system and kidneys [46]. It can develop either due to ACE2 activity on octapeptide Ang A or when the N-terminal amino acid of Ang-(1-7), Asp, is decarboxylated into Ala [47]. By binding to MrgD, alamandine boosts the cardiovascular system by, for example, counterbalancing Ang II-associated endothelial dysfunction [48].

MrgD promotes adenylyl cyclase activity, which increases cAMP levels and, in turn, protein kinase A activation and the phosphorylation of cAMP response element-binding proteins. Additionally, alamandine stimulates eNOS and the subsequent generation of NO, thereby inducing vasodilation and imposing antihypertension effects [49]. The former effect reverses vascular endothelial dysfunction and decreases the preload of the heart by aiding the venous return to the heart [50].

**Figure 2:**
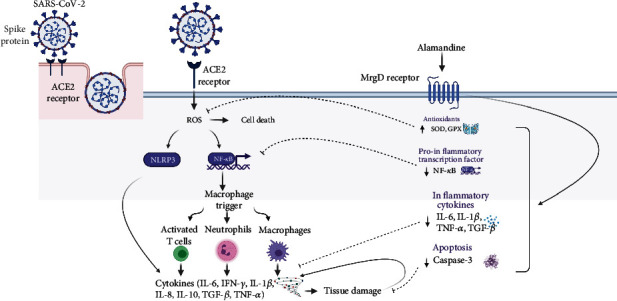
Proposed mechanisms by which alamandine inhibits the SARS-CoV-2-related cytokine storm and accompanying damage. The binding of SARS-CoV-2 to the ACE2 receptor may increase ROS production by epithelial cells. ROS, in turn, may cause cell death and increase the synthesis of NF-*κ*B and NLRP3, both of which increase cytokine levels. This phenomenon leads to immunological infiltration, which may cause illnesses such as acute respiratory distress syndrome, sepsis, and, in severe instances, death. Alamandine may mitigate these effects by increasing the levels of antioxidant enzymes (SOD, GPx) and decreasing the levels of proinflammatory cytokines (IL-1*β* and IL-6), proinflammatory transcription factor (NF-*κ*B), the profibrotic mediator (TGF-*β*), and the apoptotic factor (caspase 3). ACE2: angiotensin-converting enzyme-2; MrgD: Mas-related G protein-coupled receptor D; ROS: reactive oxygen species; NLRP3: NOD-, LRR-, and pyrin domain-containing protein 3; NF-*κ*B: nuclear factor kappa beta; IL: interleukin; SOD: superoxide dismutase; GPx: glutathione peroxidase; TGF-*β*: transforming growth factor-*β*; TNF-*α*: tumor necrosis factor *α*; IFN-*γ*: interferon-gamma.

As shown in Figure 2, various molecular pathways contribute to cytokine storms in COVID-19, resulting in multiple organ failures. The disruption of the epithelial barrier in the lungs starts a series of reactions and, eventually, cytokine storm-related damage [51]. The activation of the NLRP3 (NOD-, LRR-, and pyrin domain-containing protein 3) inflammasome and the nuclear factor kappa beta (NF-*κ*B) complex has been linked to cytokine storms [51]. The breakdown of the epithelial barrier causes bacterial infection of the lungs (or other organs) [51], and all of these activities in some way lead to oxidative stress, which is critical in the pathogenesis of viral infections [52].

Interferon gamma (IFN), transforming growth factor- (TGF-), IL-1, IL-6, IL-10, IL-8, and TNF- are among the numerous chemicals that are increased within virally mediated cytokine storms. The development of cytokine storms is one of the leading causes of mortality in people infected with SARS-CoV, MERS-CoV, and influenza [53]. Alamandine may inhibit SARS-CoV-2 infection through several possible mechanisms (Figure 2).

When given to isoproterenol-treated rats orally at a dose of 132 *μ*g/kg, alamandine-HP*β*CD (2-hydroxypropyl-*β*-cyclodextrin) had antihypertensive and antifibrotic effects. Meanwhile, when given peripherally, alamandine alleviated hypertension and the LV dysfunction associated with hypertension. Alamandine treatment (50 *μ*g/kg/day, S.C. for 42 days) blocked the PKA pathway, thereby protecting spontaneously hypertensive rats against cardiac hypertrophy and cardiomyocytes hypertrophy induced by Ang II [54].

Alamandine also possesses antifibrotic and anti-inflammatory properties. For example, alamandine (0.1 mg/kg, i.p.) increases the expression of antioxidants in the ventricles of rats that have suffered ischemia-reperfusion injuries [55]. The administration of alamandine heightened the expressions of superoxide dismutase (SOD) and catalase in ventricles affected by ischemia and reperfusion [56]. Furthermore, the expression of caspase-9, Bax, and caspase-3 in ischemia and reperfusion ventricles was also decreased by alamandine. However, when PD123319 or MrgD receptor antagonists were administered in combination with alamandine, these benefits were weakened [55].

Alamandine-HP*β*CD (30 *μ*g/kg/day, by gavage for 14 days) also lowers the effects of some proinflammatory factors (e.g., TNF-*α* and IL-1*α*) brought on by aortic constriction in mice [56]. An animal model of sepsis triggered by polysaccharides in the plasma and tissues of mice indicated increased levels of interleukin-1*β* (IL-1*β*) and TNF-*α*. At the same time, administering alamandine (1.0 *μ*M/kg, i.v.) led to lower levels of inflammatory cytokines and apoptosis in cardiac tissues [57]. It has been shown that through the MrgD receptor, alamandine reduces macrophage inflammatory responses and orients them toward an anti-inflammatory phenotype over time [58].

Alamandine (50 *μ*g/kg/day, S.C. for 42 days) also alleviated the negative proinflammatory effects of doxorubicin (DOX) (e.g., increased TNF-*α*, IL-1*β*, IL-6, TGF-*β*, and NF-*κ*B levels) [59, 60]. Moreover, a study in rats indicated that alamandine can drastically control DOX-induced cardiotoxicity and nephrotoxicity by altering rats' antioxidant status, apoptosis, and inflammatory cytokines [59]. Furthermore, Liu et al. recently showed that alamandine (2 *μ*g/kg/day, S.C. for 21 days) can inhibit bleomycin-induced lung fibrosis by reducing oxidative stress and activating autophagy through the MrgD receptor [61].

Other research shows that alamandine (50 *μ*g/kg/day, S.C. for 42 days) protects against pulmonary fibrosis and enhances the functioning of the respiratory system *in vivo* [62]. Alamandine's anti-inflammatory, antioxidant, and pulmonary protective characteristics appear to make it a promising component of SARS-CoV-2 treatments. However, further research is needed to confirm this claim and assess the risk-benefit ratio.

## 8. Conclusion

Alamandine and Ang-(1-7) are essential parts of the RAS protective arm, which possesses anti-inflammatory, antioxidative, and antifibrotic properties. In particular, the alamandine/MrgD receptor axis seems vital to protecting the body against specific diseases, including SARS-CoV-2. Although several efforts have been made to clarify the benefits of alamandine administration using animal models, such methods have not been clinically tested. Considering the crisis surrounding SARS-CoV-2 infections and related deaths, alamandine may be crucial to treating COVID*-*19 patients.

## Figures and Tables

**Table 1:** Clinical trials evaluating Ang-(1-7) as a treatment for COVID-19.

ClinicalTrials.gov identifier	Phase	Status	Study title	Study design
NCT04401423	II	Completed	TXA127 for the treatment of severe COVID-19	A double-blind, placebo-controlled randomized trial in hospitalized patients with severe COVID-19 in the United States (*n* = 22) [39]
NCT04605887	II	Recruiting	Angiotensin 1-7 as a therapy in the treatment of COVID-19	A placebo-controlled, randomized trial in hospitalized COVID-19 patients with moderate lung disease (*n* = 120) [40]
NCT04778059	II	Recruiting	Safety and efficacy of USB002 (pharmaceutically formulated Ang-(1-7)) for respiratory distress due to COVID-19	Placebo-controlled, double-blind, parallel assignment, randomized trial in hospitalized COVID-19 patients in the United States (*n* = 184) [41]
NCT04375124	Not applicable	Recruiting	Treatment of angiotensin peptide (1-7) for COVID-19	Open-label, parallel assignment, nonrandomized trial in hospitalized COVID-19 patients in Turkey (*n* = 20) [42]
NCT04633772	I/II	Recruiting	Use of angiotensin-(1-7) in COVID-19	Placebo-controlled, double-blind, parallel assignment, randomized trial in hospitalized COVID-19 in Brazil (*n* = 130) [43]
NCT04332666	II/III	Not yet recruiting	Angiotensin-(1,7) treatment in COVID-19: the ATCO trial	Placebo-controlled, double-blind, parallel assignment, randomized trial in COVID-19 patients with respiratory failure requiring mechanical ventilation in Belgium (*n* = 60) [44]
NCT04570501	I/II	Not yet recruiting	Angiotensin (1-7) for the treatment of COVID-19 in hospitalized patients	Multicenter, randomized, double-blind, placebo-controlled study in hospitalized COVID-19 patients (*n* = 160) [45]

## Data Availability

No datasets were generated or analyzed during the current study.
